# Multiplexed spatially-focused localization of light in adipose biological tissues

**DOI:** 10.1038/s41598-022-14350-3

**Published:** 2022-06-11

**Authors:** Alexander Bykov, Valery Tuchin, Igor Meglinski

**Affiliations:** 1 Opto-Electronics and Measurement Techniques, University of Oulu, Oulu, Finland; 2 Research-Educational Institute of Optics and Biophotonics, Saratov State University, Saratov, Russia; 3 Laboratory of Laser Diagnostics of Technical and Living Systems, Institute of Precision Mechanics and Control of the Russian Academy of Sciences, Saratov, Russia; 4 Interdisciplinary Laboratory of Biophotonics, Tomsk State University, Tomsk, Russia; 5 Institute of Clinical Medicine N.V. Sklifosovsky, I.M. Sechenov First Moscow State Medical University, Moscow, Russia; 6 College of Engineering and Physical Sciences, Aston University, Birmingham, UK

**Keywords:** Biophotonics, Optical physics

## Abstract

Last decades the effects of localization and focusing of light in turbid randomly inhomogeneous tissue-like scattering medium have been attracting a particular attention. Weak localization of light in disordered and weakly ordered biological tissue, polarization memory effect, correlations in transmission matrices, focusing light by wavefronts shaping have been widely exploited. Here, we represent an experimentally observed and theoretically confirmed new type of spatial localization of light within biological tissues. General description of the observed phenomenon based on Monte Carlo ray tracing model is provided. We find that innate body arrangements of individual adipocytes can act as a cascade of quasi-ordered microscale lenses confining propagation of light within adipose tissues similar to lens lightguides. The observed spatially-resolved longitudinal multi-focusing of light within disordered adipose biological tissues can naturally lead greater spatial control and enhance light-tissue interactions.

Biological tissue has complex highly heterogeneous structure with various multi-component inclusions of fractal entities exhibiting diverse spatial variations in the refractive index at the scales comparable to the wavelength of light. Interacting with such disordered medium, light is scattered dramatically, that in conjunction with a comparatively lower absorption, provides unique spectroscopic peculiarities. Scattering and diffraction of light by the structural elements of biological tissue leads to phase shifts of the light waves, and consequently to their repeated interference^1,2^. Acting as a natural shield, preventing incident light from deep penetrating in tissues, this architecture is the main obstacle to achieve a higher resolution of optical diagnostic imaging in depth. To overcome this major challenge the effects of weak localization and focusing of light through, from, or inside disordered turbid tissue-like scattering medium have been attracting a particular attention and researched intensely during the last decade^3^. Thus, weak localization of light^4^, circular polarization memory effect^5,6^, long-lived directional memory in secondary emission^7^, translation correlations^8^, transmission eigenchannels^9^, transmission matrix^10^, focusing light by wavefronts shaping^11^ in highly disordered tissue-like scattering medium have been intensively examined and widely exploited. Weak localization of light waves has been observed in quasi-two-dimensional protein nanostructures produced by silkworms^12^, and human dentin can guide light due to scattering by its tubular microstructure^13^.

Laser light generation has been explored within cellular structures, e.g. mammalian cells in an external optical cavity^14^, porcine adipocytes serving as optical resonators^15^, and human whole blood in an optofluidic ring resonator^16^. Moreover, at the microscopic level it was demonstrated that Müller cells of vertebrate retina are able to guide light from the retinal surface towards the photoreceptors^17^, whereas single human erythrocytes can serve as an adaptive lens with tuneable focus for microfluidic applications^18^. A branched flow of light with complex filament structures in the propagating field has been observed experimentally within the thin membranes^19^.

In a part of this newly emerged ‘optobiology’ paradigm^20^ we report the observation of a new type of natural spatially-resolved longitudinal multi-spot focusing localization of light within adipose tissues, where the cascade of individual adipocytes act as an ensemble of microscale lenses (Fig. 1).

**Figure 1:**
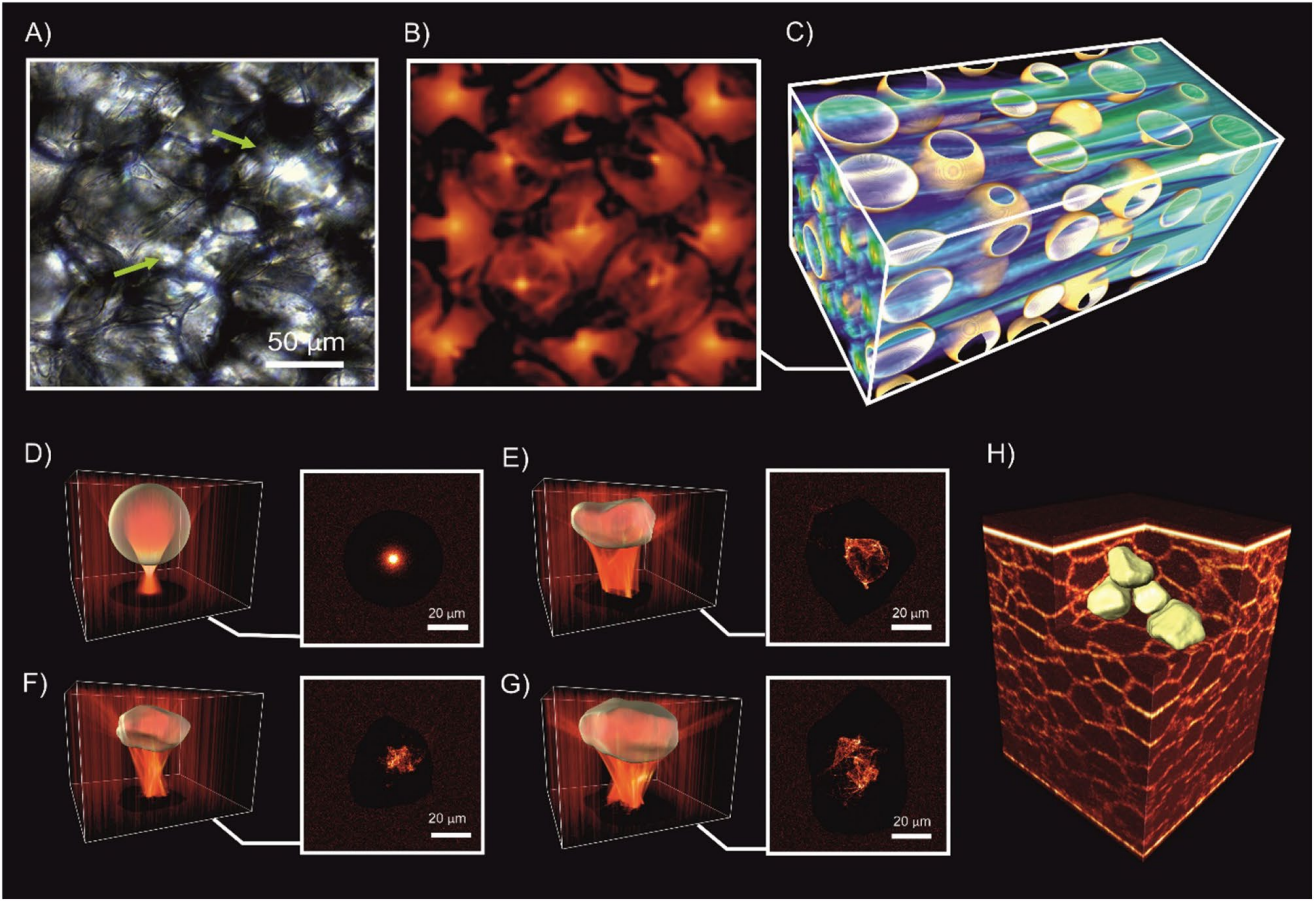
Multiplexed spatially-focused localization of light in adipose biological tissues observed experimentally (**A**), and obtained computationally (**B**) and (**C**) utilizing an idealized shape of spherical adipocytes (**D**). The shapes of individual adipocytes (**F,G**) confirmed and evaluated by the direct visualization of the porcine tissue by using optical coherence tomography (**H**).

The observation of light intensity distribution with the standard light microscopy, operating in transition mode, shows multiplexed spatially-focused localization of light (see Fig. 1A). Similar spatial distribution of the transmitted light is achieved by computational modelling (see Fig. 1B), utilized by ray tracing model (see the details in Supplementary Materials) with an idealized shape of spherical adipocytes (see Fig. 1D). The obtained results clearly show the emergence of spatially-resolved longitudinal multiplexing focusing localization of light along its propagation within adipose tissues (see Fig. 1C). While the complex shapes of the individual adipocytes are clearly imperfect lenses compare to the idealized spherical sphere (see Fig. 1D), the transmitted light field is still more tightly confined after passing through the actual adipocytes (see Fig. 1E–G). The shapes of individual adipocytes confirmed and evaluated by the direct visualization of porcine adipose tissue (see Fig. 1H) utilizing optical coherence tomography (OCT) approach^21^.

While the individual adipose cell is acting like a lens, its degree of focusing is temperature dependent. The transparency is increased with the phase transition of the lipids over the considered range of temperature and the melting of fat crystals in lipid droplets that significantly reduces scattering (22). Therefore, the observed multiplexing spatially-focused localization of light within adipose tissues reduces accordingly by the temperature decreasing, from the body one (37 °C) to the room (24 °C) value (see Fig. 2 and Supplementary materials for further details).

**Figure 2:**
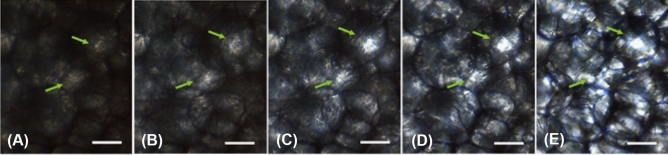
Bright-field light microscopy images of 500 µm thick porcine adipose tissue obtained at: (**A**) 24 °C, (**B**) 25 °C, (**C**) 27 °C, (**D**) 30 °C, (**E**) 37 °C. Arrows show the brightest focusing points. Scale bare corresponds to 50 µm.

## Materials and methods

### Experiment

**Figure 3:**
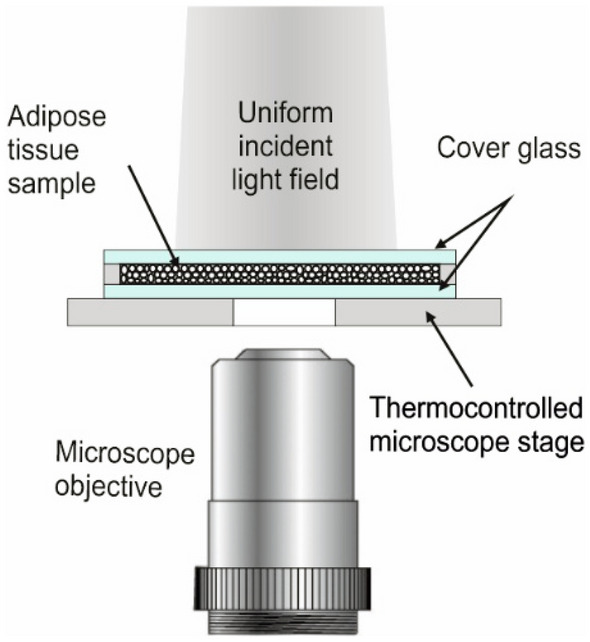
Schematic presentation of the bright-field light microscopy operating in transmittance mode in experimental measurements of adipose tissue.

Optical microscopy examination of the adipose tissue samples was performed with the standard Eclipse LV100DA-U (Nikon, Japan) microscope and 20 × objective lens in the bright-field (transmitted) mode. A thermo-control stage was used to set and maintain the temperature of the tissue sample during the study within the range of 24–44 °C (Fig. 3).

### Tissue samples

A fresh sample of porcine adipose tissue was frozen at − 25 °C. [The samples of porcine adipose tissue obtained from the local food store.] Tissue slicing was performed manually with a sharp blade. After slicing, the tissue was slowly warmed back to room temperature. The selected slice of tissue was kept at a microscope glass slide at room temperature of 24 °C prior to the measurements. The actual thickness of the sliced layer was measured with the OCT technique^23^. The thickness of the adipose tissue layer selected for the experiments was 500 µm.

### Modeling of radiative transfer through the adipose tissue

Light transport on the cellular and tissue levels was simulated using the Monte Carlo ray tracing method of statistical modelling^24^. The direction of propagation of each photon at a given time is determined by the direction unit vector $\vec{e}\left( {e_{x} ,e_{y} ,e_{z} } \right)$. The propagation directions of the photons were initialized to (0,0,1). The initial coordinates of the photons were set to ($x_{0}$,$y_{0}$,0), where $x_{0}$ and $y_{0}$ are uniformly distributed values in the *XY* plane. As such, we consider a homogeneous incident field propagating only in the *z*-direction. During propagation, the coordinates of the photon were updated according to the equations:

1 $$ \begin{array}{*{20}c} {x^{\prime} = x + e_{x} s } \\ \end{array} , $$

2 $$ \begin{array}{*{20}c} {y^{\prime} = y + e_{y} s ,} \\ \end{array} $$

3 $$ \begin{array}{*{20}c} {z^{\prime} = z + e_{z} s ,} \\ \end{array} $$

where *x*, *y* and *z* are the previous coordinate values and *s* = 10^–3^ mm is the constant step parameter determining the geometrical accuracy of the simulations.

**Figure 4:**
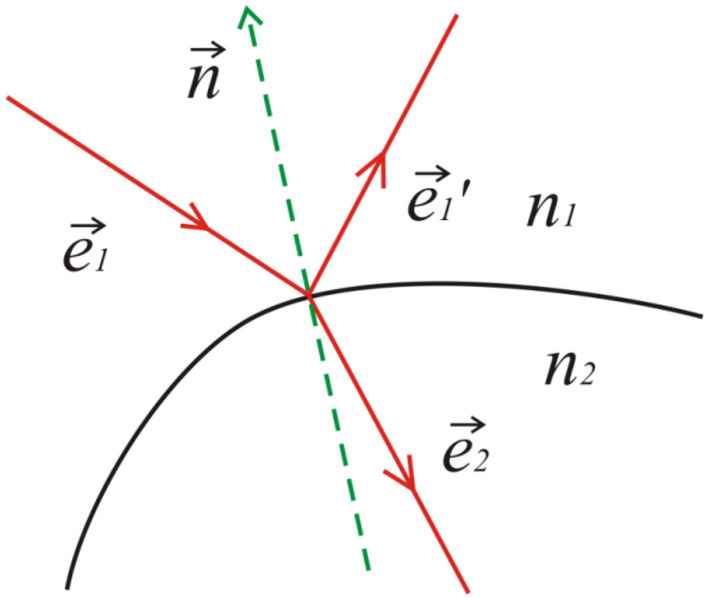
Schematic presentation of reflection and refraction of light wave at the interface of two media.

Each photon can change its propagation direction as a result of reflection or refraction at interfaces, as shown in Fig. 4. The scattering of the light within the individual adipocytes is considered as negligible.

Reflection of light in the general case is described by:

4 $$ \begin{array}{*{20}c} {\vec{e}_{1}^{^{\prime}} = \vec{e}_{1} - 2\left( {\vec{e}_{1} \cdot \vec{n}} \right)\vec{n} ,} \\ \end{array} $$

where $\vec{e}_{1}$ and $\vec{e}_{1 }^{^{\prime}}$ are the directional unit vectors of the incident and reflected beams respectively, and $\vec{n} \equiv \vec{n}\left( {n_{x} ,n_{y} ,n_{z} } \right)$ is the outer unit normal to the reflecting surface at the point of incidence (see Fig. 4). The directional vector of reflected light can be also written in a component-wise manner as:

5 $$ \begin{array}{*{20}c} {e_{1x}^{^{\prime}} = \left( {1 - 2n_{x}^{2} } \right)e_{1x} - 2n_{x} n_{y} e_{1y} - 2n_{x} n_{z} e_{1z} ,} \\ \end{array} $$

6 $$ \begin{array}{*{20}c} {e_{1y}^{^{\prime}} = - 2n_{x} n_{y} e_{1x} + \left( {1 - 2n_{y}^{2} } \right)e_{1y} - 2n_{y} n_{z} e_{1z} ,} \\ \end{array} $$

7 $$ \begin{array}{*{20}c} {e_{1z}^{^{\prime}} = - 2n_{x} n_{z} e_{1x} - 2n_{y} n_{z} e_{1y} + \left( {1 - 2n_{z}^{2} } \right)e_{1z} .} \\ \end{array} $$

In a similar way, the directional unit vector of the refracted beam $\vec{e}_{2}$ is determined by:

8 $$ \begin{array}{*{20}c} {\vec{e}_{2} = \mu \vec{e}_{1} + \left( {\sqrt {\frac{{1 - \mu^{2} }}{{\mu^{2} a^{2} }} + 1} - 1} \right)\mu a\vec{n} , } \\ \end{array} $$

where

9 $$ \begin{array}{*{20}c} {\mu = n_{1} /n_{2} } \\ \end{array} $$

and

10 $$ \begin{array}{*{20}c} {a = \left( {\vec{e}_{1} \cdot \vec{n}} \right) = e_{1x} n_{x} + e_{1y} n_{y} + e_{1z} n_{z} . } \\ \end{array} $$

$n_{1}$ and $n_{2}$ are the refractive indices on either side of the interface respectively (see Fig. 4). A component-wise representation in this case gives:

11 $$ \begin{array}{*{20}c} {e_{2x} = \mu e_{1x} + \left( {\sqrt {\frac{{1 - \mu^{2} }}{{\mu^{2} a^{2} }} + 1} - 1} \right)\mu an_{x} , } \\ \end{array} $$

12 $$ \begin{array}{*{20}c} {e_{2y} = \mu e_{1y} + \left( {\sqrt {\frac{{1 - \mu^{2} }}{{\mu^{2} a^{2} }} + 1} - 1} \right)\mu an_{y} , } \\ \end{array} $$

13 $$ \begin{array}{*{20}c} {e_{2z} = \mu e_{1z} + \left( {\sqrt {\frac{{1 - \mu^{2} }}{{\mu^{2} a^{2} }} + 1} - 1} \right)\mu an_{z} .} \\ \end{array} $$

The amount of light reflected and refracted is accounted for in accordance with the Fresnel formula for unpolarized radiation):

14 $$ \begin{array}{*{20}c} {R\left( {\alpha_{i} } \right) = \frac{1}{2}\left[ {\frac{{\sin^{2} (\alpha_{i} - \alpha_{t} )}}{{\sin^{2} (\alpha_{i} + \alpha_{t} )}} + \frac{{\tan^{2} (\alpha_{i} - \alpha_{t} )}}{{\tan^{2} (\alpha_{i} + \alpha_{t} )}}} \right] ,} \\ \end{array} $$

where *α*_*i*_ is the angle of incidence of the photon and *α*_*t*_ is the angle of refraction.

For the simulation, the 3D tissue model, presented in Fig. 1C, was formed of a set of quasi-regularly packed spheres with the diameter uniformly distributed within the range 60 ± 10 µm, consistent with a typical porcine adipose tissue^25^. The spheres were considered as optically soft particles with a relative refractive index *m* = 1/*µ* = 1.03 typical for biotissues^26^. A total of 10^6^ individual rays were used for the simulation. The absorption and scattering by the lipid constituents of the fat cells at 37 °C were considered to be negligibly small.

For the modelling of the focusing effects of individual cells, presented in Fig. 1E–G, cell shapes were extracted from the 3D Optical Coherence Tomography (OCT) image, presented in Fig. 1H. The boundaries of several representative adipose tissue cells were manually segmented in a layer-by-layer manner from the 3D OCT images using Matlab software. When simulating the light-focusing ability of the individual cells, the relative index of refraction was set to *m* = 1.44 (see Ref.^27^), corresponding to the case when the cell is placed in the air.

Finally, the adipose tissue is formed of aggregated clusters of adipocytes. The main role of these cells is containing stored fat in the form of droplets of lipids (predominantly triglyceride) in a semi-liquid state at the body temperature. We find that innate body arrangements of the individual adipocytes can act as a cascade of quasi-ordered microscale lenses confining propagation of light within the adipose tissue similar as in lens lightguides. The observed multiplexing spatially focused localization of light within disordered adipose biological tissues can naturally lead greater spatial control of the wavefront shaping and enhanced light-tissue interactions.

## Supplementary Information

Supplementary Video 1.

Supplementary Information 1.

## Data Availability

All data generated or analysed during this study are included in this published article and its supplementary information files.
