# Supplementary material for: Multiplexed spatially-focused localization of light in adipose biological tissues
Source: Sci Rep. 2022 Jun 11;12:9711. doi: 10.1038/s41598-022-14350-3 (PMC9188595; doi:10.1038/s41598-022-14350-3)
Supplement: Supplementary file 2 — Supplementary Information 1. [file 41598_2022_14350_MOESM2_ESM.docx]

**Supplementary**

**Multiplexed spatially-focused localization of light in adipose biological tissues**

Alexander Bykov^*^, Valery Tuchin, and Igor Meglinski

^*^ Correspondence to: [alexander.bykov@oulu.fi](mailto:alexander.bykov@oulu.fi)

In addition to conventional microscopy approach the standard spectral-domain hyperion OCT imaging system (Thorlabs, USA) with a central wavelength of 930 nm providing 5.8 µm (axial) and 8 µm (lateral) resolution (in air) was used for the characterization of the adipose tissue. Tissue samples were placed on a heating plate with controlled temperature. During the measurements, the temperature of the sample was continuously increased from the room temperature of 24 °C to 50 °C.

Fig.2 in the main text of the paper shows the series of images of an enhancement of the radiative focusing transfer obtained during the heating. These results are well agreed with the results of alternative measurements utilizing OCT^1^. The corresponding OCT images of the adipose tissue slice observed during the heating for 24 °C, 30 °C and 37 °C are presented in Fig.S1-(A, B, C) and can be seeing at the Supplementary Movie 1. Due to the great increase of the transparency of individual lipocytes caused by heating, the forward focusing radiation transfer through these cells is amplified significantly, whereas the backscattering of light from the adipose tissue cells progressively decreases. Therefore, the transparent adipose cells become looking darker in the OCT images (see Fig.S1 and the Supplementary Movie).


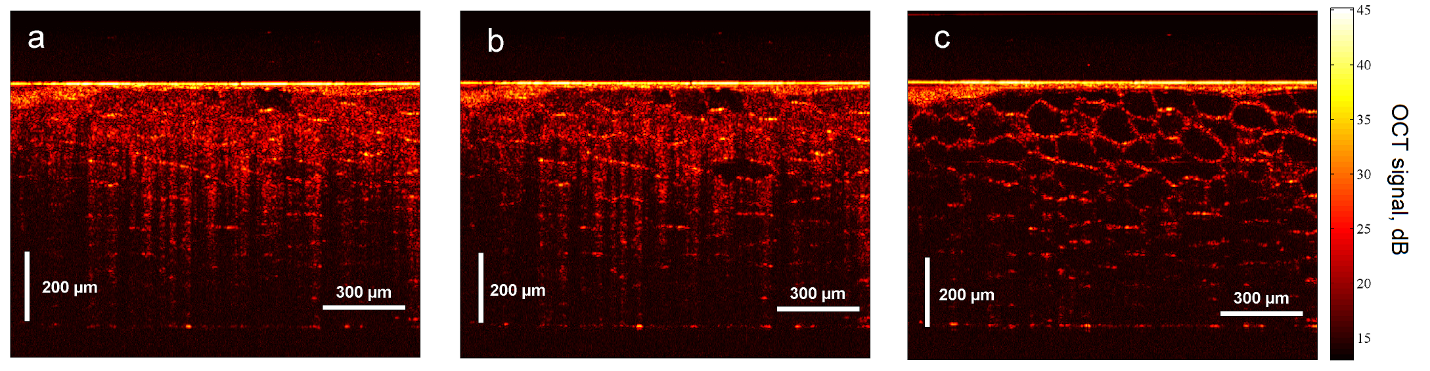


**Figure S1.** Selected OCT images of the 500 µm thick porcine adipose tissue slice obtained at (A) 24°C, (B) 30°C and (C) 37°C.

Fig.S2 shows the relative changes of the intensity of OCT signal measured at the arbitrary selected topical subsurface area (5.8 µm × 5.8 µm × 8 µm) of the adipose tissue sample along the temperature increase. As one can see strong (over 5 times) decrease of the intensity of OCT signal is observed between 24 and 37°C. This is due to the phase transition of the lipids in frame of the considered range of temperature that enhances significantly transparency with the individual adipose cells^2^ with the corresponding increase of focusing radiation transfer.

**Figure S2.** An intensity of OCT signal at the arbitrary topical subsurface area (5.8 µm × 5.8 µm × 8 µm) of the adipose tissue sample during the temperature increase from 24 to 50 °C. The standard deviation is not exceeding 2.5%.

References:

1. A. Bykov, T. Hautala, M. Kinnunen, A. Popov, S. Karhula, S. Saarakkala, M.T. Nieminen, V.V. Tuchin, and I. Meglinski, Imaging of subchondral bone by optical coherence tomography upon optical clearing of articular cartilage, *J. Biophoton*. **9**(3), 270 – 275 (2016).
2. I. Yanina, A. Popov, A. Bykov, I. Meglinski, and V. Tuchin, Monitoring of temperature-mediated phase transitions of adipose tissue by combined optical coherence tomography and Abbe refractometry. *J. Biomed. Opt.* **23**(1), 016003 (2018).

**Captions for the Supplementary Movie:** The recorded OCT images of the 500 µm adipose tissue slice during the heating from 24 °C to 37 °C.
